# Bacterial Community Structures in Freshwater Polar Environments of Svalbard

**DOI:** 10.1264/jsme2.ME16074

**Published:** 2016-09-30

**Authors:** Spyridon Ntougias, Żaneta Polkowska, Sofia Nikolaki, Eva Dionyssopoulou, Panagiota Stathopoulou, Vangelis Doudoumis, Marek Ruman, Katarzyna Kozak, Jacek Namieśnik, George Tsiamis

**Affiliations:** 1 Department of Environmental Engineering, Democritus University of ThraceXanthiGreece; 2 Department of Analytical Chemistry, The Chemical Faculty, Gdansk University of TechnologyGdanskPoland; 3 Department of Environmental and Natural Resources Management, University of PatrasAgrinioGreece; 4 Faculty of Earth Sciences, University of Silesia, Centre for Polar Studies KNOW (Leading National Research Centre)SosnowiecPoland

**Keywords:** cryophilic microorganisms, psychrophiles, polar freshwater environments, stream and lake water, 16S rRNA gene pyrosequencing

## Abstract

Two thirds of Svalbard archipelago islands in the High Arctic are permanently covered with glacial ice and snow. Polar bacterial communities in the southern part of Svalbard were characterized using an amplicon sequencing approach. A total of 52,928 pyrosequencing reads were analyzed in order to reveal bacterial community structures in stream and lake surface water samples from the Fuglebekken and Revvatnet basins of southern Svalbard. Depending on the samples examined, bacterial communities at a higher taxonomic level mainly consisted either of *Bacteroidetes*, *Betaproteobacteria*, and *Microgenomates* (OP11) or *Planctomycetes*, *Betaproteobacteria*, and *Bacteroidetes* members, whereas a population of *Microgenomates* was prominent in 2 samples. At the lower taxonomic level, bacterial communities mostly comprised *Microgenomates*, *Comamonadaceae*, *Flavobacteriaceae*, *Legionellales*, SM2F11, *Parcubacteria* (OD1), and TM7 members at different proportions in each sample. The abundance of OTUs shared in common among samples was greater than 70%, with the exception of samples in which the proliferation of *Planctomycetaceae*, *Phycisphaeraceae*, and *Candidatus* Methylacidiphilum spp. lowered their relative abundance. A multi-variable analysis indicated that As, Pb, and Sb were the main environmental factors influencing bacterial profiles. We concluded that the bacterial communities in the polar aquatic ecosystems examined mainly consisted of freshwater and marine microorganisms involved in detritus mineralization, with a high proportion of zooplankton-associated taxa also being identified.

Several extreme environments are populated by a broad diversity of microorganisms that live at low temperatures, even below the pure water freezing point (6). These cold environments constitute an unexhausted source of vast metabolic potential that may be linked to the impact of climate change and used to exploit cold-active enzymes for biotechnological applications (5, 14).

Ambient cold environments are widely distributed on earth, including the alpine zones of the highest mountain ranges of the world, the deep ocean, the Arctic Circle, tundra, and Antarctica (44). Cold-adapted soil ecosystems must cope with wide temperature fluctuations and often radiation, whereas deep ocean microbes live at low, albeit constant temperatures (13). The prevailing low temperature and/or low liquid water availability in these ecosystems are considered to be major limiting factors for growth (44, 53). However, psychrophiles overcome these severe conditions through complex adaptive mechanisms. Increased membrane fluidity at lower temperatures (59), the accumulation of compatible osmolytes and secretion of anti-freezing proteins (16, 26), the induction of cold-shock proteins (32), and conformational flexibility of psychrophilic enzymes (56) are among the main adaptive strategies adopted by the cryophilic microbiota.

Polar zones, including Svalbard, are strongly affected by prolonged temperature increases in the globe, which are incommensurate in these regions and have led to the accelerating retreat of glaciers (34, 63). Nutrient fluxes from various sources, *i.e.* aeolian and guano inputs (60, 61), glacial flour particle release (39), and the transport of entrapped organic and inorganic compounds through cryoconite holes (61), influence in a complex matter biogeochemical cycles as well as the size and structure of microbial communities in the Arctic cryosphere (50). Interactions between ice-covered land and marine ecosystems (25), and freshwater from retreating glaciers (21, 62) also contribute to the Arctic microbial food web. On the other hand, cold ecosystems may act as ecological screens for immigrant microbiota, with the latter dealing with severe environmental conditions, such as successive freeze/thaw events, high surface radiation, and low nutrient and liquid water availability (25).

Members of the phyla *Bacteroidetes*, *Actinobacteria*, and *Firmicutes* together with the *Alpha-*, *Beta-*, and *Gamma*-subdivisions of *Proteobacteria* are the predominant taxa identified in cryophilic environments (27, 40, 62). The fjords and archipelago of Svalbard have recently attracted attention for assessing the impact of anthropogenic activities and global warming on the microbial ecology of the European part of the Arctic (20, 39, 57, 61). Microbial communities and glacial run-off to ice lakes have been extensively examined; however, information on the structures of microbial communities in the southern part of the Spitsbergen/Svalbard island complex is limited.

In the present study, the main objective was to characterize bacterial communities in the Fuglebekken and Revvatnet basins, two neighboring catchments located in geologically, hydrologically, and phytosociologically diverse regions of Svalbard, through pyrosequencing, and to link these bacterial profiles with various abiotic environmental factors.

## Materials and Methods

### Sampling procedure and site description

**Fig. 1:**
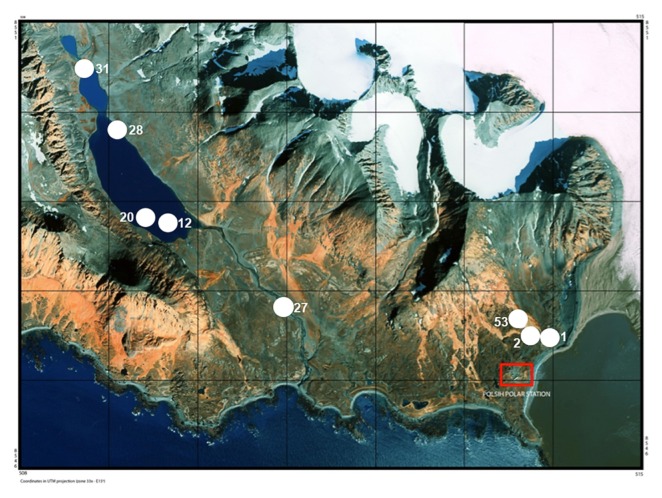
Sampling site map (Kolondra L., Norway. Svalbard, Spitsbergen, Orthophotomap 1:10,000, NPI-Tromsø & University of Silesia). Sample S1 (FS): stream water (77 00.351, 15 33.203); Sample S2 (FL): lake (77 00.390, 15 32.928); Sample S53 (FL): lake (77 00.455, 15 32.928); Sample S20 (RL): lake (77 01.106, 15 22.401); Sample S27 (RR): river (77 01.630, 15 26.265); Sample S28 (RS): stream (77 01.662, 15 20.919), Sample S31 (RL): lake (77 01.989, 15 20.919). The Polish Polar Station is indicated in red.

Surface water was collected from eight sites located in the Fuglebekken and Revvatnet basins between the 9th and 13th of August, 2010. Freshwater samples were obtained from the Fuglebekken stream (S1 [FS]), the Fuglebekken Lake (S2 [FL] and S53 [FL]), the Revvatnet lake (S12 [RL], S20 [RL], and S31 [RL]), the Revvatnet stream (S28 [RS]), and the Revelva river (S27 [RR]) (Fig. 1). Field research work did not include endangered or protected species and sampling permits were provided by The Governor of Svalbard. The Fuglebekken and Revvatnet basins, which drain to the Hornsund fjord, are situated in the southern part of the island of Spitsbergen and their substratum belongs to the Hecla Hoek Precambrian formations (28). Geographical coordinates are presented in Fig. 1, while a detailed description of the sampling sites is provided in Table S1.

All water samples (3×0.5 L each, 1.5 L in total) were collected from a depth of 20 to 50 cm into sterile polyethylene bottles and maintained at low temperatures (4°C) in the absence of preservatives, as described in detail by Larose *et al.* (35). In the streams, sampling sites were chosen in sections of fast, turbulent, flow, with care to avoid contamination from the disturbed bed sediment. Sample temperatures ranged between 0.8 and 1.6°C.

### Chemical analysis

The pH and electrical conductivity (EC) of freshwater samples were assessed using an Elmetron CX-401 apparatus supported by an ESAgP-301W electrode and CD-2-conductivity meter. Standards for pH and EC calibrations were purchased from Metrohm. Solid particles were retained by filtration (0.45 μm, Millipore) where necessary.

A range of organic compounds were analyzed in the Department of Analytical Chemistry (Faculty of Chemistry) of Gdansk University of Technology: total anionic, cationic, and non-ionic surfactants, total phenols, and formaldehyde were measured spectrophotometrically (SQ 118, PHARO 100, Merck) according to Spectroquant methods. Total organic carbon was measured using the CM 5300 furnace (UIC Coulometrics) supplemented with a coulometric detector (CM 5014 CO_2_ Coulometer). The TOC device was operated under a high mineralization temperature (950°C), at which oxygen was served as the carrier gas at a flow rate of 100 mL min^−1^.

Anions and cations were analyzed in the Polish Polar Station (St. Siedlecki, Hornsund) using ion chromatography (IC 761 Compact, Metrohm). Anions and cations were measured using Metrosep A Supp 5 and Metrosep C 4 (Metrohm) chromatography columns (150×4.0 mm each), with sodium carbonate (3.2 mM)-sodium hydrogen carbonate (1.0 mM) and HNO_3_ (1.7 mM)-dipicolinic acid (0.7 mM) serving as eluents at flow rates of 0.7 and 0.9 mL min^−1^, respectively. Injection volumes were 20 μL and 100 μL for anions and cations, respectively. All standards (1,000 mg L^−1^ each), eluents, and reagents for the spectrophotometric analyses were purchased from Merck (Darmstadt, Germany). 18.2 MΩ deionized water was obtained using the Millipore Gradient A10 water purification system (Bedford, USA).

An analysis of metals was performed using inductively coupled plasma mass spectrometry (ICP-MS, Elan DRC, PerkinElmer). The operating parameters of ICP-MS are listed below: sample uptake rate, 1 mL min^−1^; nebulizer gas flow rate, 0.98 L min^−1^; plasma gas flow, 15 L min^−1^; RF power, 1,300 W.

### Genomic DNA extraction and 16S rRNA gene pyrosequencing

In order to isolate genomic DNA, 0.5-L water samples were successively filtered through sterile filters of decreasing porosities (3-μm and 0.2-μm glass fiber and membrane filters, respectively, Whatman). Total DNA was extracted as previously described in Katsaveli *et al.* (33) and quantified with a Qubit fluorometer (Invitrogen). In brief, 1.2 mL of 10% w/v sodium dodecyl sulfate (SDS) and 175 μL of 20 mg mL^−1^ proteinase K were added to the filter and incubated at 37°C under periodic stirring. Four milliliters of 5 M NaCl and 3.3 mL of preheated 2% w/v cetyltrimethylammonium bromide (CTAB) solution (2% w/v CTAB, 1.4 M NaCl, 100 mM Tris-HCl, and 20 mM EDTA, pH 8) were added. Samples were then incubated at 65°C for 2 h and an equal volume of chloroform/isoamyl alcohol (24:1 [v/v]) was added for phase separation. Samples were subsequently centrifuged at 6,000×*g* for 30 min and the aqueous phase was collected. DNA was precipitated by adding an equal volume of isopropanol and centrifuging at 10,000×*g* at 4°C for 20 min. DNA was washed twice with 2 mL 70% v/v ethanol, left to dry in a vacuum desiccator, and dissolved in 200 μL of sterile Milli-Q water. The concentrations of the isolated DNA samples were: S1_FS: 25 ng μL^−1^, A260/A280=1.75; S2_FL: 42 ng μL^−1^, A260/A280=1.79; S53_FL: 38 ng μL^−1^, A260/A280=1.86; S12_RL: 67 ng μL^−1^, A260/A280=1.72; S20_RL: 54 ng μL^−1^, A260/A280= 1.67; S31_RL: 86 ng μL^−1^, A260/A280=1.81; S28_RS: 33 ng μL^−1^, A260/A280=1.83; S27_RR, 28 ng μL^−1^, A260/A280=1.68.

Bacterial diversity in stream and lake surface water samples was assessed by tag-encoded FLX amplicon pyrosequencing in 50 μL PCR reaction containing 100 ng genomic DNA each. The universal primers 926F (5′-CTYAAAKGAATTGRCGG-3′) and 1392R (5′-ACGGGCGGTGTGTRC-3′) were used to amplify the ~470-bp region of the bacterial 16S rRNA gene (47). PCR was performed through an initial denaturation period at 94°C for 3 min, followed by 32 cycles consisting of DNA denaturation at 94°C for 30 s, primer annealing at 55°C for 40 s, and an elongation step at 72°C for 1 min, with a final termination reaction at 72°C for 5 min. Amplicons from different samples were merged in equal concentrations and cleaned up using Agencourt Ampure beads (Agencourt Bioscience Corporation). Samples were sequenced using Roche’s 454 FLX titanium instrument technologies based on the manufacturer’s guidelines.

### Sequence and statistical analyses

Sequences were analyzed using the QIIME package (10). Briefly, all sequences from a single pyrosequencing run entered a custom QIIME pipeline after denoising. Sample IDs were allocated using a mapping file and barcodes were assigned to each sample. Sequences were excluded from the analysis if they were less than 200 bp in size, the quality score was less than 25, they contained ambiguous characters or an uncorrectable barcode, or did not include the primer sequence. 16S rRNA gene sequences were clustered using uclust (19) and assigned to operational taxonomic units (OTUs) with a similarity threshold of 97%. Representative sequences from each OTU were selected and aligned via Pynast (11). Taxonomy was assigned using the SILVA111 16S rRNA gene database.

Based on square root-transformed read abundance data, a Bray-Curtis similarity matrix was calculated (9). Overall similarities in microbial communities were displayed using a multidimensional scaling plot (MDS) and tests on the multivariate null hypothesis were performed using the non-parametric statistical test PERMANOVA (2). Significant relationships among environmental factors and bacterial profiles were examined using distance-based multivariable (DISTLM) and redundancy (dbRDA) analyses. Analyses were performed using the PERMANOVA+ plugin option through PRIMER6 (3).

### Data accession

The gene sequences obtained have been deposited in EMBL under study accession number PRJEB13414.

## Results and Discussion

The physicochemical analysis revealed that samples S2 (FL) and S53 (FL) showed the highest pH, EC, TOC, anion, and cation values as well as the highest Cu, Se, Sr, Sb, and U concentrations among the stream and lake surface freshwaters examined (Table 1). In a comparison with the other aqueous specimens tested, sample S12 (RL) contained the highest Mn, Mo, Ni, and Zn concentrations and some of the highest As, Ba, and Rb concentrations (Table 1).

**Fig. 2:**
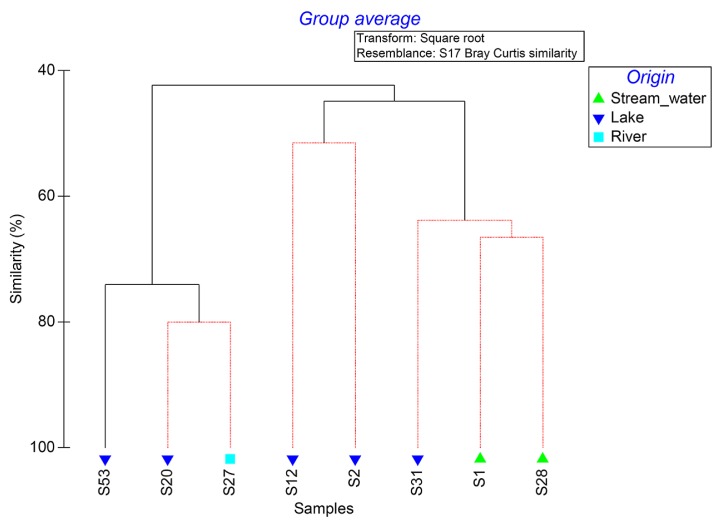
Clustering analysis of bacterial community structures of stream and lake surface water samples obtained from Fuglebekken and Revvatnet basins. Clusters drawn in red/black branches indicate significant/non-significant relationships.

A total of 52,928 sequences (average length of 458 nucleotides) were obtained after the quality assessment. Samples S1 (FS) and S28 (RS) showed the highest bacterial diversity, while samples S2 (FL), S12 (RL), and S20 (RL) were the least diverse (Table 2). A clustering analysis of sequence data revealed that none of the samples examined were identical (Fig. 2). However, samples from Lakes Revvatnet and Fuglebekken together with sample S27 (RR), which was from a river fed with water from Lake Revvatnet, were more likely to be grouped together. The other group was comprised of the stream water samples S28 (RS) and S1 (FS) as well as sample S31 (RL), which was obtained nearby the delta of the Revelva river (Fig. 2).

**Fig. 3:**
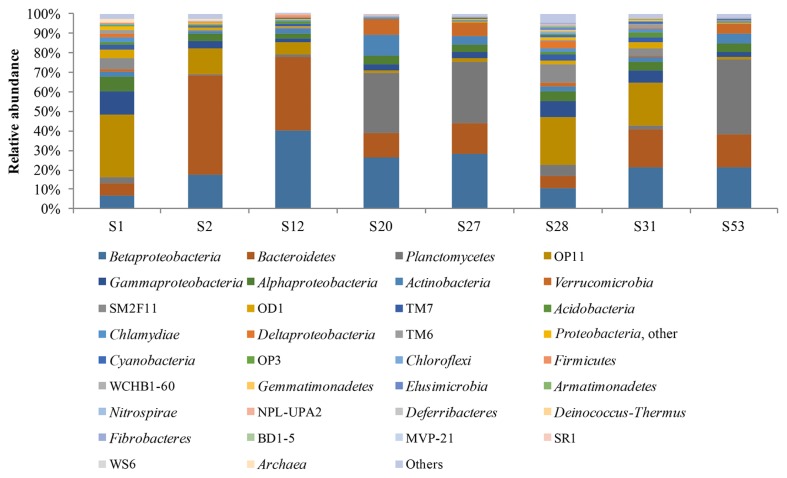
Bacterial community structures and relative abundances (based on the number of pyrosequencing reads) of major taxa identified in stream and lake surface water samples obtained from Fuglebekken and Revvatnet basins (Grouping is at the phylum level, except for *Proteobacteria*).

Samples S2 (FL), S12 (RL), and S31 (RL) were dominated by members of the phylum *Bacteroidetes*, the class *Betaproteobacteria*, and the *Microgenomates* (OP11) division, followed by the taxa of *Alphaproteobacteria* and *Gammaproteobacteria* (Fig. 3). However, the predominant taxa in these samples, *i.e. Bacteroidetes*, *Betaproteobacteria*, and *Microgenomates*, showed proportional differences (Fig. 3). In samples S20 (RL), S27 (RR), and S53 (FL), *Planctomycetes*, *Betaproteobacteria*, and *Bacteroidetes* were the major microbiota, followed by *Actinobacteria*, *Alphaproteobacteria*, *Gammaproteobacteria*, and *Verrucomicrobia* (Fig. 3). The dominance of *Planctomycetes* has not been previously identified in Arctic environments using universal primers; however, it was recently detected as a minor microbial component of the Arctic benthos (54, 55, 62). In a study performed in the Eastern Alps, *Planctomycetes* was identified as the predominant group in the microbiome of the arctic-alpine lichen *Solorina crocea* (23). The predominance of *Microgenomates* members was revealed in samples S1 (FS) and S28 (RS), followed by the lower abundances of the *Alpha-*, *Beta-*, and *Gamma-*subdivisions of *Proteobacteria*, *Bacteroidetes*, *Planctomycetes*, and candidate phylum SM2F11 (Fig. 3). The *Microgenomates* (OP11) division has rarely been reported in cold environments, *e.g.* in the polygonal tundra soils of Siberia (36), and mainly as a minor component of microbial diversity. Since *Microgenomates* was also among the main taxa identified in samples S2 (FL), S12 (RL), and S31 (RL), the ecological role of this microbial group appears to be significant in the biogeochemical cycles occurring in Arctic Circle habitats. This is the first study to show a dominant *Microgenomates* community in polar ecosystems. The *Microgenomates* and *Parcubacteria* communities were the main microbiota in Pavin Crater Lake (France), in which their possible involvement in sulfur and iron cycling was highlighted (8). Moreover, candidate division SM2F11 was also represented in an important proportion (up to 9% for sample S28 [RS]), which is among the highest reported in the literature. However, the function of this bacterial group remains unknown.

**Fig. 4:**
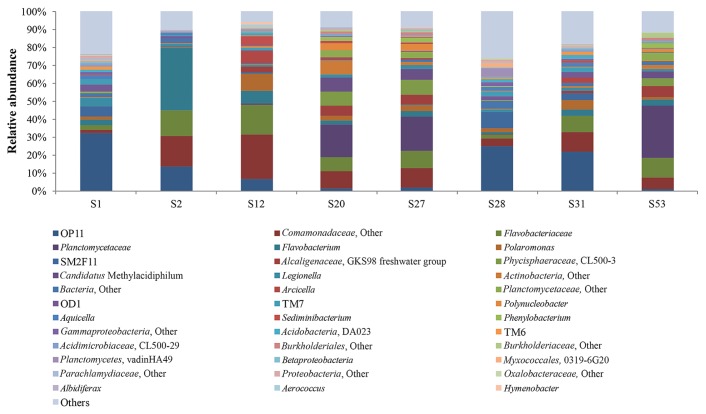
Relative abundance, based on the number of pyrosequencing reads, of major bacterial taxa in stream and lake surface water samples obtained from Fuglebekken and Revvatnet basins.

At the lower taxonomic levels, less than 20 taxa dominated in all the samples analyzed, with the sum of their abundances being in the range of 72–89% (Fig. 4 and Table 3). The most abundant taxa in samples S20 (RL), S27 (RR), and S53 (FL) were *Planctomycetaceae*, *Comamonadaceae*, *Flavobacteriaceae*, *Phycisphaeraceae*, *Alcaligenaceae*, and *Candidatus* Methylacidiphilum spp., while members of *Microgenomates*, *Comamonadaceae*, and *Flavobacteriaceae*, at various abundances, were the main taxa in the other samples examined (Fig. 4 and Table 3). The bacterial communities in the Fuglebekken and Revvatnet basins mainly comprised *Microgenomates*, *Comamonadaceae*, *Flavobacteriaceae*, *Legionellales*, SM2F11, *Parcubacteria*, and TM7 members as well as two unclassified bacterial and actinobacterial linkages, but at different proportions in each. The abundance of taxa that were shared in common among samples was greater than 70%, with the exception of samples S20 (RL), S27 (RR), and S53 (FL) in which the proliferation of *Planctomycetaceae*, *Phycisphaeraceae*, and *Candidatus* Methylacidiphilum members reduced the relative abundance of all OTUs shared in common (ranging in these samples between 33 and 38%) (Table 3). Skidmore *et al.* (52) showed that microbial populations and community structures in polar freshwater ecosystems are influenced by their aqueous geochemistries. *Comamonadaceae* and *Flavobacteriaceae* spp. were previously reported to be abundant in oligotrophic freshwater environments, including the Canadian High Arctic glaciers (15). *Flavobacterium*-rich communities have also been identified in Antarctic freshwater habitats (38), suggesting their possible involvement in the bacterioplankton mineralization process (1, 38). Similar to *Flavobacteria*, *Planctomycetaceae* and *Phycisphaeraceae* spp. are common inhabitants of detrital aggregates (22), which are linked to algal blooms and the degradation of algal sulfated polysaccharides (43). *Planctomycetes* has also been proposed to be indirectly involved in chemolithotrophic iron oxidation because of its prominent presence in the iron-hydroxide deposits of volcanic hydrothermal vents (54).

Apart from the freshwater and marine microorganisms that were predominant in the samples examined, zooplankton-associated taxa were also identified. *Legionellales* members, *i.e. Legionella*, *Coxiella*, and *Aquicella* spp., comprised an important part of the bacterial communities in the Fuglebekken and Revvatnet basins. *Legionellales* spp. are potential intracellular parasites of free-living protozoa (12, 48, 51) and have been reported previously in polar environments, exhibiting host specificity (12). *Chlamydiales*, including *Neochlamydia (*29), *Candidatus* Metachlamydia (18), *Candidatus* Protochlamydia (41), and *Candidatus* Rhabdochlamydia spp. (17), and *Rickettsiales*, such as *Rickettsia*, *Holospora*, *Caedibacter*, *Candidatus* Captivus, and *Candidatus* Odyssella spp. (4, 7, 49), are obligate intracellular bacteria represented by nine and eleven OTUs, respectively, in all samples analyzed. The proportion of these taxa varied widely across sites and ranged between 1.4% (sample S12 [RL]) and 12.2% (sample S1 [FS]). Thus, endosymbiosis appears to be an important strategy in cryophilic microbiota of the Arctic and a mechanism to be adapted in polar temperatures (12).

The taxa identified also provide evidence for the biogeochemical pathways possibly occurring in the ecosystems of the Fuglebekken and Revvatnet regions. In the nitrogencycling process, the absence of the nitrite-oxidizing *Nitrobacter* spp. was notable. Nitrifiers were not detected in sample S27 (RR) or S53 (FL), whereas *Nitrosomonadaceae* and *Nitrospirales* spp. were the only ammonia and nitrite-oxidizing taxa identified in other samples, showing relatively low abundances (0.1–0.5% each of these functional groups). No nitrite oxidizers were identified in sample S2 (FL). *Nitrospirae* rather than proteobacterial nitrite oxidizers appears to be involved in the complete oxidation of ammonia in the Arctic cryosphere, and this is supported by the experimental findings of previous studies (30, 46, 55). Despite *Candidatus* Brocadia being a minor component of the microbial population in a single sample and no other known anammox bacteria being identified, the predominance of *Planctomycetes* in some of the samples does not exclude the presence of novel microbiota capable of removing nitrogen through anammox in the oxygen-deficient zones of polar environments.

Furthermore, sulfur and iron oxidation may have occurred in the stream and lake surface water samples analyzed because sulfur/sulfide and/or iron oxidizers, *i.e. Gallionellaceae*, *Acidimicrobiaceae*, *Acidiferrobacter*, *Beggiatoa*, *Sulfuritalea*, and *Thiobacillus* spp., were identified in most samples (with the exception of sample S2 [FL]), but at low abundances (0.1–1%, depending on the site examined). Filamentous sulfur bacteria of the genus *Beggiatoa* appear to play an important ecological role in the Arctic archipelago of Svalbard (31). Moreover, microbial ferrous oxidation was recently found to contribute to macroscopic aggregate formation in aquatic ecosystems (37). Dissimilatory sulfate reduction appears to be restricted because only a single OTU related to an *Albidiferax* sp. was detected in most samples (apart from S28 [RS]).

**Fig. 5:**
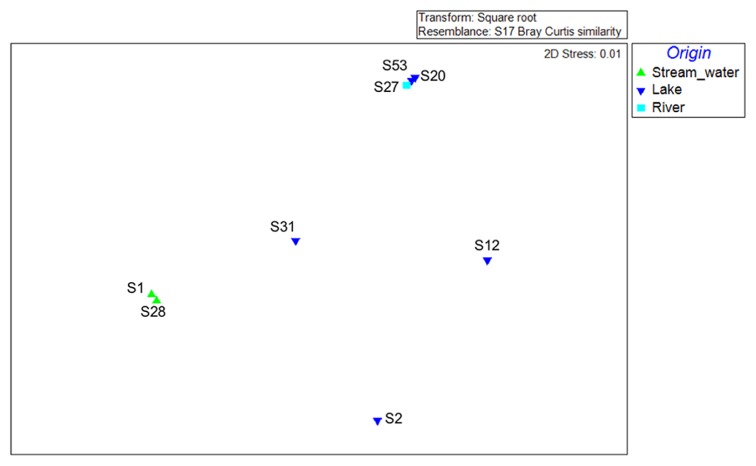
Multidimensional scaling (MDS) plot for a graphical illustration of changes in the bacterial community structure. Green triangles represent stream water samples, Inverted blue triangles represent lake samples, Cyan squares represent rivers samples. Similarities in ribotype profiles were calculated by the Brey-Curtis algorithm.

**Fig. 6:**
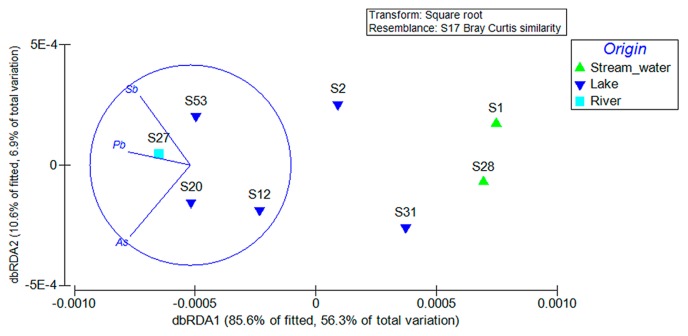
dbRDA within a DistLM analysis of environmental factors. The fitted variation was predicted by the linear model adopted in the DistLM analysis, whereas the total variation referred to the original measurements.

MDS was used to establish whether bacterial community structures changed as a function of the origin of the samples (lake, river, and stream water). MDS ordinations are typically interpreted based on the distance among ordinate points, with treatments that appear close together representing samples with similar bacterial community compositions. MDS ordinations based on Bray-Curtis similarities are illustrated in Fig. 5. Samples from the lake and the river fed with lake water were similar, while samples from stream water formed a separate distinct cluster (PERMDISP, F=35.529, P[perm]= 0.017). The stress values on the ordination plots were less than 0.01, indicating that the plots observed are realistic representations of the data obtained. A multivariable analysis (DISTLM) was performed in order to examine the influence of various environmental factors on the bacterial community composition (Table 1). Among the 39 factors examined, only As (*p*<0.05), Pb (*p*<0.05), and Sb (*p*<0.05) had a significant influence. The primary axis of dbRDA (Fig. 6) describes 56.3% of the total variation, but 85.6% of the variation within the linear model created by DistLM to analyze environmental variables (Fig. 6). Based on the dbRDA plot, increases in the concentration of As affected sampling sites S20 (RL) and S12 (RL), while Sb and Pb affected sampling sites S27 (RR) and S53 (FL). Despite S12 (RL) being relatively distinct in its bacterial composition with lake and river samples S20 (RL), S27 (RR), and S53 (FL) (Fig. 2–4), they formed a tight cluster as a consequence of their similar As, Sb, and Pb concentrations (Fig. 6).

Increases in As, Sb, and Pb concentrations appeared to negatively affect the *Microgenomates* population. *Microgenomates (*OP11) is not commonly found in heavy metal-contaminated environments. In samples S20 (RL), S27 (RR), and S53 (FL), *Planctomycetes* was more abundant than *Microgenomates*. *Planctomycetes* is an important constituent of As- and Pb-rich habitats (45), and is among the most active bacterial taxa in As-contaminated ecosystems (24). Moreover, an important *Polaromonas* and *Flavobacterium*-like population was detected in sample S12 (RL), which was recently found among the major microbiota in As-rich habitats (42, 58).

## Conclusion

Wide bacterial diversity of an aquatic origin was detected, which appears to be involved in detritus mineralization processes. Moreover, a dominant *Microgenomates* population was identified in some of the polar freshwater ecosystems examined. This is the first study on a dominant *Microgenomates* community in polar ecosystems, and the ecological role of this microbial group in the biogeochemical cycles occurring in Arctic Circle habitats warrants further investigation. Based on the abiotic factors examined, only As, Pb, and Sb appear to have had significant effects on the structures of microbial communities in arctic samples. A high proportion of zooplankton-associated taxa was also identified, indicating that endosymbiosis is an adaptive mechanism by several microorganisms in the Arctic cryosphere.

## Supplementary Information



## Figures and Tables

**Table 1:** Physicochemical characteristics of stream and lake surface water samples obtained from Fuglebekken and Revvatnet basins

Characteristic	S1 (FS)	S2 (FL)	S12 (RL)	S20 (RL)	S27 (RR)	S28 (RS)	S31 (RL)	S53 (FL)
pH	7.92±0.05	9.45±0.05	7.01±0.07	7.11±0.06	7.06±0.07	7.05±0.07	7.09±0.06	9.48±0.05
EC (μS cm^−1^)	127±7.5	144±7.8	49.1±0.98	55.2±0.97	57.5±0.98	30.7±0.99	49.3±0.99	146±8.1
TOC (mg L^−1^)	82.1±0.88	88.7±0.84	56.5±0.92	50.2±0.99	58.9±0.89	27.1±0.98	39.7±0.96	89.2±0.81
Total cationic surfactants (mg L^−1^)	0.12±0.065	0.10±0.065	0.05±0.005	<0.05	0.07±0.005	0.09±0.05	<0.05	0.07±0.05
Total anionic surfactants (mg L^−1^)	0.06±0.005	0.08±0.005	0.11±0.055	0.07±0.005	0.10±0.055	0.14±0.055	0.17±0.055	0.06±0.005
Total non-ionic surfactants (mg L^−1^)	<0.1	<0.1	<0.1	<0.1	<0.1	<0.1	<0.1	<0.1
Formaldehyde (mg L^−1^)	<0.02	<0.02	<0.02	0.04±0.007	0.05±0.006	<0.02	0.05±0.006	0.05±0.006
Total phenols (mg L^−1^)	<0.025	<0.025	<0.025	<0.025	<0.025	<0.025	<0.025	<0.025
Anions								
F^−^ (mg L^−1^)	0.01±0.0088	0.01±0.0089	<0.01	<0.01	<0.01	<0.01	<0.01	0.01±0.0093
Cl^−^ (mg L^−1^)	6.16±0.055	10.2±1.4	5.31±0.059	5.42±0.055	6.09±0.051	3.51±0.061	4.08±0.049	10.6±0.44
NO_2_^−^ (mg L^−1^)	<0.01	0.014±0.0075	<0.01	<0.01	<0.01	<0.01	0.010±0.0054	0.013±0.0062
NO_3_^−^ (mg L^−1^)	1.05±0.087	6.01±0.066	0.19±0.0061	0.17±0.0072	0.35±0.0037	0.17±0.0065	0.25±0.0045	6.53±0.071
HCO_3_^−^ (mg L^−1^)	62±7.9	62±7.8	12±9.7	11±9.9	12±9.6	7.3±3.6	22±9.1	62±8.1
SO_4_^2−^ (mg L^−1^)	6.92±0.33	6.02±0.35	3.40±0.51	3.69±0.55	3.98±0.48	1.18±0.76	4.38±0.41	6.30±0.31
Cations								
Na^+^ (mg L^−1^)	3.64±0.44	5.70±0.36	3.16±0.47	3.20±0.43	3.76±0.44	2.13±0.51	2.51±0.44	5.76±0.37
K^+^ (mg L^−1^)	0.59±0.031	0.62±0.027	0.48±0.039	0.38±0.044	0.56±0.031	0.17±0.071	0.82±0.022	0.80±0.021
NH_4_^+^ (mg L^−1^)	<0.01	<0.01	0.01±0.008	<0.01	0.01±0.009	<0.01	<0.01	<0.01
Mg^2+^ (mg L^−1^)	0.99±0.023	1.84±0.55	0.73±0.031	0.62±0.041	0.88±0.036	0.40±0.052	0.89±0.038	1.87±0.61
Ca^2+^ (mg L^−1^)	22.2±2.31	22.7±2.45	4.44±0.64	4.35±0.62	5.14±0.49	2.29±0.88	7.27±0.33	22.6±2.4
Metals								
Ag (μg L^−1^)	<0.001	<0.001	<0.001	<0.001	<0.001	<0.001	<0.001	<0.001
As (μg L^−1^)	<0.001	0.08±0.0045	0.21±0.041	0.20±0.044	0.22±0.028	0.11±0.041	0.20±0.044	0.20±0.049
Ba (μg L^−1^)	2.52±0.42	2.91±0.45	5.20±0.33	5.01±0.37	4.90±0.29	1.11±0.59	8.81±0.21	3.91±0.38
Cd (μg L^−1^)	0.051±0.0044	0.051±0.0041	<0.001	<0.001	<0.001	<0.001	<0.001	<0.001
Co (μg L^−1^)	<0.001	<0.001	<0.001	<0.001	<0.001	<0.001	<0.001	<0.001
Cs (μg L^−1^)	<0.001	<0.001	<0.001	<0.001	<0.001	<0.001	<0.001	<0.001
Cu (μg L^−1^)	0.40±0.051	0.52±0.044	0.30±0.041	0.21±0.041	0.50±0.022	<0.001	0.41±0.0032	0.71±0.0025
Mn (μg L^−1^)	<0.001	<0.001	1.01±0.31	0.23±0.052	0.61±0.051	0.11±0.062	0.40±0.051	<0.001
Mo (μg L^−1^)	0.11±0.053	0.12±0.049	0.15±0.051	0.11±0.055	0.14±0.051	<0.001	0.22±0.049	0.13±0.053
Ni (μg L^−1^)	<0.001	<0.001	0.61±0.033	<0.001	0.091±0.0033	<0.001	<0.001	<0.001
Pb (μg L^−1^)	0.021±0.0039	0.031±0.0029	0.031±0.0028	0.050±0.0020	0.041±0.0019	<0.001	<0.001	0.030±0.0021
Rb (μg L^−1^)	0.16±0.049	0.18±0.044	0.37±0.031	0.36±0.029	0.26±0.039	0.32±0.029	0.44±0.021	0.19±0.042
Sb (μg L^−1^)	0.021±0.0048	0.042±0.0033	0.030±0.0040	0.031±0.0030	0.051±0.0039	0.021±0.0051	0.022±0.0053	0.060±0.0031
Se (μg L^−1^)	0.27±0.041	0.29±0.043	0.11±0.052	<0.001	0.21±0.048	0.15±0.050	0.16±0.051	0.30±0.041
Sr (μg L^−1^)	48.3±2.1	50.3±1.9	12.3±3.1	21.0±2.3	22.5±2.7	10.1±3.9	12.1±3.3	60.8±1.9
Sn (μg L^−1^)	<0.001	<0.001	<0.001	<0.001	<0.001	<0.001	<0.001	<0.001
Th (μg L^−1^)	0.034±0.0051	0.029±0.0055	<0.001	0.011±0.0069	0.012±0.0071	<0.001	0.011±0.0068	0.018±0.0066
Tl (μg L^−1^)	<0.001	<0.001	<0.001	<0.001	<0.001	<0.001	<0.001	<0.001
U (μg L^−1^)	0.101±0.049	0.105±0.050	<0.001	<0.001	0.034±0.0077	<0.001	<0.001	0.108±0.053
Zn (μg L^−1^)	0.71±0.023	0.31±0.055	1.31±0.23	<0.001	0.81±0.024	<0.001	<0.001	<0.001

Values are presented as the mean±standard deviation (mean±STDEV).

**Table 2:** Species richness and diversity among 16S rRNA gene pyrosequencing libraries

Sample	Number of reads^1^	Species diversity indices	

		Simpson	Shannon
S1 (FS)	10,567	86.90±0.99	6.34±0.03
S2 (FL)	1,912	39.50±1.15	4.13±0.06
S12 (RL)	2,783	42.20±1.02	4.71±0.04
S20 (RL)	15,759	44.10±1.18	4.89±0.05
S27 (RR)	7,620	51.70±1.25	5.22±0.04
S28 (RS)	5,417	87.07±1.00	6.38±0.02
S31 (RL)	4,788	76.00±0.89	5.96±0.04
S53 (FL)	4,082	48.30±1.24	5.03±0.06

1 Operational taxonomic units (OTUs) were defined at a 97% sequence identity threshold.

**Table 3:** Major bacterial taxa identified in Fuglebekken and Revvatnet basins

Taxon	Sample (relative abundance in %)							

	S1 (FS)	S2 (FL)	S12 (RL)	S20 (RL)	S27 (RR)	S28 (RS)	S31 (RL)	S53 (FL)
*Microgenomates* (OP11)§	**32.2**	**13.4**	**6.6**	**1.1**	**1.8**	**24.6**	**21.7**	0.9
*Comamonadaceae*, Other§	**1.7**	**17.1**	**24.8**	**9.7**	**10.7**	**4.7**	**10.9**	**6.6**
*Flavobacteriaceae*§	**2.6**	**14.2**	**16.6**	**7.8**	**9.8**	**1.9**	**9.2**	**10.8**
*Planctomycetaceae*	0.2	0	0.6	**18.3**	**18.9**	0.4	0.1	**29**
*Flavobacterium*§	**3.1**	**34.8**	**7.2**	**2.2**	**3**	0.9	**3.6**	**3.6**
*Polaromonas*§	**1.5**	0.4	**9.2**	**2.7**	**3.8**	**2.3**	**5.2**	**1.2**
Candidate division SM2F11§	**5.9**	0.7	0.9	0.1	0.1	**9.2**	**3.8**	0.2
*Alcaligenaceae*, GKS98 freshwater group	0	0	**3**	**5.4**	**5.2**	0	**1.1**	**5.9**
*Phycisphaeraceae*, CL500-3	0	0	0.3	**8**	**8.4**	0	0	**4.6**
*Candidatus* Methylacidiphilum	0	0	0	**7.7**	**6.1**	0	0	**4**
*Legionella*§	**4.8**	**1.3**	0.7	**2**	**2**	**1.5**	**2.1**	**1.2**
*Actinobacteria*, Other§	0.1	0.4	0.5	**7.6**	**1.9**	0.4	0.6	**2**
*Bacteria*, Other§	**2.4**	**2.4**	0.3	0.6	**1.1**	**4.3**	**1.6**	**1.7**
*Arcicella*	0.1	0	**6.9**	**1**	**1.2**	0	**3.2**	0.4
*Planctomycetaceae*, Other	0.7	0	0.1	**4**	**3.3**	0.3	0.1	**4.9**
*Parcubacteria* (OD1)§	**4**	**1.3**	0.3	0.1	0.3	**2.3**	**3**	0.3
Candidate division TM7§	**2.8**	0.4	**1.6**	0.2	0.1	**2.6**	**2.8**	0.2
*Polynucleobacter*	0	0	**1**	**3.8**	**3.8**	0	0.2	**1.9**
*Aquicella*§	**2.6**	**1**	0.2	0.2	0.3	**2.5**	**2.2**	0.1
*Sediminibacterium*	0	0	**5.4**	**1.1**	0.9	0	**1.4**	0.1
*Phenylobacterium*	0.1	0	0.3	**2.2**	**2.5**	0.1	0.1	**2.4**
*Gammaproteobacteria*, Other§	**1.5**	0.3	0.3	0.5	0.3	**2.4**	**1**	0.3
*Acidobacteria*, DA023§	**1.2**	0.5	**1.5**	0	0.1	**1.6**	**2.1**	0.1
Candidate division TM6§	**1.9**	0.3	0.4	0.1	0.3	0.8	**1.6**	0.7
*Acidimicrobiaceae*, CL500-29§	0.9	0.1	0.8	**1**	0.8	0.9	0.6	0.6
*Burkholderiales*, Other	0	0	0.2	**1.3**	**1.4**	0.1	0.1	**1.5**
*Burkholderiaceae*, Other	0	0	0.1	**1.1**	**1.1**	0	0	**2.7**
*Planctomycetes*, vadinHA49	0.5	0	0	0	0	**4.2**	0.4	0
*Betaproteobacteria*, Other§	**1.2**	0.1	0.4	0.1	0.2	0.7	**1**	0.1
*Myxococcales*, 0319-6G20	0.8	0	0	0	0	**2.3**	0.4	0
*Parachlamydiaceae*, Other§	**1.2**	0.4	0.2	0	0.2	0.8	0.6	0
*Proteobacteria*, Other§	**1.1**	0.4	0.3	0.1	0.2	0.9	0.2	0
*Oxalobacteraceae*, Other§	0.7	0.1	0.4	0.1	0.1	**1.1**	0.7	0.3
*Albidiferax*	0	0.1	0.1	**1.1**	0.7	0	0.1	0.1
*Aerococcus*	0	0	**1.5**	0.2	0	0	0	0
*Hymenobacter*	0	0	**1.1**	0	0.1	0	0	0

Others	24	10	6	9	9	26	18	12

Number of taxa with an abundance >1%*	17	8	13	20	18	15	18	16

* marked with bold-type case.

§ indicates taxa that were detected in all samples examined with an abundance >1% in at least one sample (20 phylogenetic linkages). Five more linkages with an abundance <1% (*Chitinophagaceae*, *Pedobacter*, and *Sandarakinorhabdus* spp. within the order *Sphingobacteriales*, a WCHB1-60 taxon, and a *Coxiella* sp. within the order *Legionellales*) were also detected in all samples of the present study.
