# Supplementary material for: Bacterial Community Structures in Freshwater Polar Environments of Svalbard
Source: Microbes Environ. 2016 Sep 30;31(4):401–9. doi: 10.1264/jsme2.ME16074 (PMC5158112; doi:10.1264/jsme2.ME16074)
Supplement: Supplementary file 1 [file 31_401_s1.pdf]

Table S1. Description of sampling sites.

Site description
**Sampling site:** Fuglebekken<br>**Sample designations (Geographic coordinates: latitude, longitude):**<br>sample S1: stream water (77 00.351, 15 33.203)<br>sample S2: lake (77 00.390, 15 32.928)<br>sample S53: lake (77 00.455, 15 32.928)<br>**Fuglebekken site description:**<br>The Lake Fuglebekken covers an area of ca 2-3 km^2 and is surrounded by a major part of the Fuglebergsletta plain and the steep slopes of the Ariekammen and Fugleberget. The main lake stream is directed to Isbjørnhamna bay [Wang *et al.* 2014. Ecotoxicology 23:1890–1899].<br>Three distinct periods influence the hydrology of the Fuglebekken lake: fast outflow until middle of July owing to snow melting, moderate flows owing to late summer rainfalls, and massive flows owing to intensive winter precipitation [Wang *et al.* 2014. Ecotoxicology 23:1890–1899; White *et al.* 2000. Antarct. Sci. 12:386–393].<br>The basin is composed by metamorphic rocks of the Ariekammen mountain ridge, in the proterozoic Isbjørnhamna formation. Slates of marble, biotite granite and carbonate gneisses are the main constituents of the formation, where Ariekammen and Fugleberget slopes are subjected to intensive weathering [Wang *et al.* 2014. Ecotoxicology 23:1890–1899].
**Sampling site:** Revelva river and Lake Revvatnet<br>**Sample designations (Geographic coordinates: latitude, longitude):**<br>sample S12: lake (77 01.083, 15 23.056)<br>sample S20: lake (77 01.106, 15 22.401)<br>sample S27: river (77 01.630, 15 26.265)<br>sample S28: stream (77 01.662, 15 21.767)<br>sample S31: lake (77 01.989, 15 20.919)<br>**Revelva site description:**<br>The Revelva river forms a primary path of 5.3 km which passes through the lake. The catchment is defined by the ridges Brattegga (645 m), Skoddefjellet (733 m) and Torbjørnsenfjellet (663 m), and the Gulf of Ariebukta. The headwaters of the largest streams are located in the upper part of the catchment, on Eimfjellet (640 m) and Skålfjellet (635 m) slopes. Due to catchment asymmetry, left bank streams are predominant, with Ariebekken stream, which is originated from the Arie glacier.<br>Revelva river discharges at the Ariebukta Gulf (Hornsund fjord), where oceanic water goes back at a distance of approximately 100 m at high tide [Xiang *et al.* 2009. Soil Sci. Plant Nutr. 55:523–531].<br>Revelva valley comprises of rocks belonging to the Precambrian formations Hecla-Hoek, which is formed by the crystalline schists and marbles of the Ariekammen formations and the crystalline schists blue-mica of Skoddefjellet, in the group of Isbjørnhamna rock. A part of the basin nearby Revelva springs belongs to the Eimfjellet group geological formation, which includes the Gulliksenfjellet quartzites, the Torbjørnsenfjellet amphibolites and the metamorphic rocks of Gangpasset [Zarsky *et al.* 2013. Environ. Res. Lett. 8:035044].<br>Revelva springs are located among sheepbacks, and a bottom moraine can be found at Skålfjellet slopes. The lower part of the valley consists of an abrasive inselberg with frost crack areas and of storm shafts [Zeng *et al.* 2013. Arch. Microbiol. 195:313–322]. A large mesotrophic moist moss-tundra has been identified by Polish scientists [Zeng *et al.* 2009. Polar Biol. 32:1447–1460], including *Calliergon sarmentosum*, *Tetraplodon mnioides* and *Saxifraga hyperborea* communities. Moreover, a dominant *Ranunculus spitsbergensis* community was detected at the bottom of the valley. Several algae were found in the standing water of marshes, in the streams and in the lake Revvatnet.
